# Mega-dose sodium ascorbate: a pilot, single-dose, physiological effect, double-blind, randomized, controlled trial

**DOI:** 10.1186/s13054-023-04644-x

**Published:** 2023-10-12

**Authors:** Fumitaka Yanase, Sofia Spano, Akinori Maeda, Anis Chaba, Thummaporn Naorungroj, Connie Pei Chen Ow, Yugeesh R. Lankadeva, Clive N. May, Ashenafi H. Betrie, Darius J. R. Lane, Glenn M. Eastwood, Mark P. Plummer, Rinaldo Bellomo

**Affiliations:** 1 Department of Intensive Care, Austin Hospital, Melbourne, VIC Australia; 2 Australian and New Zealand Intensive Care Research Centre (ANZIC-RC), School of Public Health and Preventive Medicine, Monash University, Melbourne, Australia; 3 The Florey Institute of Neuroscience and Mental Health, The University of Melbourne, Melbourne, VIC Australia; 4 Department of Intensive Care, Royal Adelaide Hospital, Adelaide, Australia; 5 Department of Critical Care, University of Melbourne, Melbourne, Australia; 6 Data Analytics Research and Evaluation Centre, Austin Hospital, Melbourne, Australia; 7 Department of Intensive Care, Royal Melbourne Hospital, Melbourne, Australia

**Keywords:** Sodium ascorbate, Vitamin C, Sepsis, Septic shock, Vasopressors, Sequential organ failure score

## Abstract

**Background:**

Mega-dose sodium ascorbate (NaAscorbate) appears beneficial in experimental sepsis. However, its physiological effects in patients with septic shock are unknown.

**Methods:**

We conducted a pilot, single-dose, double-blind, randomized controlled trial. We enrolled patients with septic shock within 24 h of diagnosis. We randomly assigned them to receive a single mega-dose of NaAscorbate (30 g over 1 h followed by 30 g over 5 h) or placebo (vehicle). The primary outcome was the total 24 h urine output (UO) from the beginning of the study treatment. Secondary outcomes included the time course of the progressive cumulative UO, vasopressor dose, and sequential organ failure assessment (SOFA) score.

**Results:**

We enrolled 30 patients (15 patients in each arm). The mean (95% confidence interval) total 24-h UO was 2056 (1520–2593) ml with placebo and 2948 (2181–3715) ml with NaAscorbate (mean difference 891.5, 95% confidence interval [− 2.1 to 1785.2], *P* = 0.051). Moreover, the progressive cumulative UO was greater over time on linear mixed modelling with NaAscorbate (*P* < 0.001). Vasopressor dose and SOFA score changes over time showed faster reductions with NaAscorbate (*P* < 0.001 and *P* = 0.042). The sodium level, however, increased more over time with NaAscorbate (*P* < 0.001). There was no statistical difference in other clinical outcomes.

**Conclusion:**

In patients with septic shock, mega-dose NaAscorbate did not significantly increase cumulative 24-h UO. However, it induced a significantly greater increase in UO and a greater reduction in vasopressor dose and SOFA score over time. One episode of hypernatremia and one of hemolysis were observed in the NaAscorbate group. These findings support further cautious investigation of this novel intervention.

*Trial registration* Australian New Zealand Clinical Trial Registry (ACTRN12620000651987), Date registered June/5/2020.

**Supplementary Information:**

The online version contains supplementary material available at 10.1186/s13054-023-04644-x.

## Introduction

Sepsis is common in the intensive care unit (ICU) [1]. It accounts for approximately 35–50% of in-hospital deaths worldwide [2] and, when associated with shock, it carries a high mortality [3–5]. High dose Vitamin C (ascorbic acid at 6 g/day or more) has been prescribed as an adjunctive therapy for sepsis because of its antioxidant and anti-inflammatory effects [6–8] and the frequent presence of vitamin C hypovitaminosis [9]. Randomized controlled trials, however, have shown variable results or even possible harm [10–12]. Moreover, current guidelines and a Bayesian re-analysis of a previous trial have led to recommendations not to use intravenous vitamin C in patients with sepsis because of possible harm [13, 14]. Regrettably, these studies used ascorbic acid, a compound associated with the induction of metabolic acidosis and acidemia [15]. This makes it uncertain whether the unclear effects in human sepsis were due to the administration of a suboptimal preparation of the study medication. In contrast, sodium ascorbate (NaAscorbate) has a physiological pH, does not contribute to acidosis, can be given at a much greater dose, and may carry a different efficacy profile [16].

NaAscorbate has recently been studied in pre-clinical experiments in a sheep model of septic shock [16]. In these experiments, mega-dose NaAscorbate increased urine output (UO), renal medullary tissue perfusion and oxygenation, and significantly reduced the vasopressor dose required to maintain target blood pressure. The mode of action of NaAscorbate in protecting the kidney in septic shock is unknown. However, we have shown that NaAscorbate reverses renal medullary hypoxia in sheep with septic shock [16]. Medullary hypoxia appears to a central pathway to mediating renal dysfunction and injury in the setting of sepsis [17, 18]. We hypothesized that such medullary renal protection might have also occurred during sepsis in humans.

Accordingly, we conducted a pilot, single-dose, double-blind, randomized controlled trial to evaluate the physiological effect of a single mega-dose of NaAscorbate compared to placebo in patients with septic shock. We aimed to test the primary hypothesis that mega-dose NaAscorbate would significantly increase the total UO in the first 24 h compared with placebo. We also aimed to test the secondary hypotheses that NaAscorbate would increase the cumulative UO over time and decrease the vasopressor dose requirements needed to achieve target blood pressure and would also lower the sequential organ failure assessment (SOFA) score.

## Methods

### Trial design and ethical oversight

This study was a pilot, single-dose, single-center, double-blind, and randomized controlled trial conducted in a tertiary hospital in Melbourne, Australia. Ethics approval was obtained from Austin Health Human Research Ethics Committee (HREC/64579/Austin-2020, approval date: September, 17, 2020) and the trial was registered with the Australian New Zealand Clinical Trial Registry (ACTRN12620000651987) before the start of enrolment.

Written informed consent for enrolment or consent to continue and use patient data was obtained from all patients or the legally responsible person.

### Patients

We studied adult patients in intensive care unit (ICU) within 24 h of septic shock onset [1]. Exclusion criteria are listed in detail in Additional file 1. Key exclusion criteria included pregnancy, imminent death, hemodialysis, glucose-6 phosphate dehydrogenase (G-6PD) deficiency, or suspected history of oxalate nephropathy.

### Protocol amendments

Because a patient (12th patient) with chronic kidney injury (CKD) developed a high plasma sodium level (161 mEq/L) and elevated ketone measured by point of care device (subsequently measured by central laboratory and found to be falsely elevated) after the first 50 g of NaAscorbate infusion, we subsequently excluded patients with a creatinine level > 150 µmol/L and/or a Na level > 155 mEq/L before enrolment. In addition, after the 13th patient, we allowed enrolment of patients, who were not for cardiopulmonary resuscitation (CPR) but was for all other treatments (renal replacement therapy, mechanical ventilation and vasopressors). As this study was not designed to assess mortality, but to assess the physiological effects of NaAscorbate and to study feasibility and safety for a future phase II trial, we considered that if the patient was not for CPR but was for all other treatments, enrolment was justified.

### Randomization

We randomly assigned eligible patients to the NaAscorbate group or placebo, with a 1:1 ratio using a computer-generated random number allocation system with permuted blocks and opaque, sealed envelopes.

### Rationale for the choice of intervention and dose

The dose of NaAscorbate used in this pilot study was less than that used in experimental animals [16]. However, it was chosen as a first cautious step in a program dedicated to the methodical and systematic investigation of mega-dose NaAscorbate therapy in sepsis. The dose was also based on previous experience. During the COVID-19 pandemic, a patient had been treated with 60 g of NaAscorbate locally on compassionate grounds. Such patient had shown improvements in vasopressor support and no side effect.^14^

### Intervention

Patients in the NaAscorbate group received 30 g of NaAscorbate Solution (30 g in 100 ml, Biological Therapies, Melbourne, Australia) diluted in 150 ml 5% glucose solution over 1 h followed by a further 30 g diluted with 150 ml 5% glucose over 5 h (60 g over 6 h in total). Patients in the control group received a fluid volume-matched 5% glucose solution (vehicle), administered over 1 h followed by 250 ml 5% glucose over 5 h. Research staff who were independent from clinical care prepared the study drug. The study drug was identical and labelled as “Vitamin C 30 g or Placebo in 250 ml 5% glucose”. The study drug (NaAscorbate or placebo) was infused via a central venous catheter. All research data were extracted from electronic medical record (EMR) system by the research staff. All EMR data were entered by bedside nurses or doctors who did not know study allocation. Also, the laboratory team who measured all biomarkers and/or NaAscorbate levels were blinded to treatment allocation.

We collected arterial blood gas data at baseline, one, four and six h after commencement of the infusion. If the blood gas sodium value increased more than 10 mmol/L from the baseline or to an absolute sodium value > 160 mmol/L, the study drug was immediately ceased. This threshold was amended from the initial protocol to a change of > 7 mmol/L or an absolute Na value > 155 mmol/L from the 13th patient. All other aspects of septic shock treatment were dictated by the treating ICU specialists.

### Blood sampling

Blood samples were collected into ethylenediaminetetraacetic acid (EDTA) tubes at baseline, 1, 4 and 6 h after commencement of the infusion for the measurement of inflammatory markers and vitamin C. Blood was centrifuged immediately for 10 min (3000 rpm) with plasma then stored in Eppendorf tubes at − 80 °C. An additional 300 µL of plasma was immediately added to a solution containing 1.2 mL of MeOH/H2O (90:10, v/v) and 313 µM diethylenetriaminepentaacetic acid (DETAPAC). The mixture was then centrifuged for 10 min at 8000 rpm and resultant supernatant was stored at − 80 °C. This facilitated the deproteination and chelation of metals in the plasma samples, which would otherwise interfere with measurement of ascorbate levels.

### Ascorbate and biomarker measurement

The concentration of ascorbate in the processed plasma was determined fluorometrically using an end-point kinetic microplate assay adapted from assays developed by Lane et al. and Vislisel et al. for determination of ascorbate in cultured cells [19, 20].

Plasma levels of inflammatory chemokines IL-6, IL-8, IL-10, vascular endothelial growth factors alpha, and C-reactive protein were measured by commercially available ELISA kits (ThermoFisher Scientific, Melbourne, Australia).

### Study outcomes

The primary outcome was the total urine output in the 24 h after commencement of the study drug infusion. All patients had an indwelling urinary catheter and their urinary output was recorded in the EMR by bedside nurses every hour. Such nurses were blinded to treatment allocation.

Other secondary outcomes included changes in progressive cumulative UO, vasopressor requirements and sequential organ failure assessment (SOFA) score over time; time alive and free of vasopressors at day seven after study drug commencement, and alive ICU-free day. If a patient died, they were assigned zero alive and vasopressor-free or ICU-free days.

Other outcomes included mortality at ICU discharge and hospital discharge, hospital length of stay, plasma ascorbate levels and inflammatory markers. We also assessed urine samples for the presence of urine oxalate crystals before the commencement of study drug infusion and at 24 h.

### Sample size

We based our estimated effect on the findings of sheep experiments. In such sheep experiments, we observed a urinary output increase from 10 to 500 ml/hr during the infusion of 3.75 g/kg over 6 h [16]. We assumed that after the cessation of the infusion UO would be equivalent between placebo and NaAscorbate [16]. The dose we administered was one fourth of the dose given to sheep. This would imply that the difference in UO would be approximately 125 ml/hr. Over 6 h, this would equal 750 ml. However, humans have no capacity to generate vitamin C, while sheep can make endogenous vitamin C. Thus, we assumed a somewhat greater effect than seen in the sheep and rounded off the expected effect to 950 ml [16]. We also assumed that UO may be a non-parametric distribution and we added a 20% inflation factor. We calculated that inclusion of 15 patients in each group would have an 80% power to detect a mean (standard deviation [SD]) difference in total 24-h UO of 950 (800) ml at an alpha level of 0.05.

### Statistical analysis

Descriptive statistics were used to summarize patient characteristics for each group. Continuous variables were reported as median with interquartile range (IQR) and categorical variables were reported as proportions. Vasopressors were converted to norepinephrine equivalent dose according to previously published conversion tables [21, 22].

The primary outcome was analyzed using a *t*-test to compare mean urine output between the two groups at 24 h after the assessment of normality by the histogram, the *Q*–*Q* plots, and the Shapiro–Wilk’s test. The equality of variances was assessed by an *F*-test (Additional file 1: Fig. S1). For secondary outcomes, categorical variables were analyzed using chi-squared tests or Fisher’s exact tests as appropriate. For continuous variable, we robustly estimated the median difference between the groups by a non-parametric bootstrapping procedure. This technique generated 10,000 bootstrap samples by randomly resampling our original data with replacement. For each sample, we calculated the median for both groups and the difference between these two values. This process generated a distribution of 10,000 bootstrapped median differences from which we could construct a 95% confidence interval. We calculated bias-corrected and accelerated (BCa) confidence intervals to account for any potential bias or skewness in the bootstrap distribution.

As a sensitivity analysis, we built linear mixed models with time as random effect to study the interaction with group allocation for cumulative urinary output, norepinephrine equivalent dose, serum sodium, serum creatinine, serum lactate and SOFA. For these variables, the evolution within the first 24 h was expressed graphically as change from baseline. Statistical significance was defined as a *P* value less than 0.05. Due to the exploratory nature of the study no *P* value adjustments were applied for multiple comparisons. The normality of the residual distribution was assessed by histogram and *Q*–*Q* plots (Additional file 1: Fig. S2).

To assess serum biomarker changes over time for the two treatment groups, we fitted linear mixed models including fixed effects for time and treatment group as well as random effect for the intercept and treatment group nested within the time variable. The random effect structure was specified using a diagonal covariance matrix. To accommodate the nonlinear relationship for serum sodium ascorbate, we transformed the variable using a natural logarithm transformation. The model assumptions, including linearity and normality of residuals, were checked using diagnostic plots.

The statistical analysis was performed using R software version 4.2.2 (R foundation, Vienna, Austria).

## Results

### Baseline characteristics

**Fig. 1:**
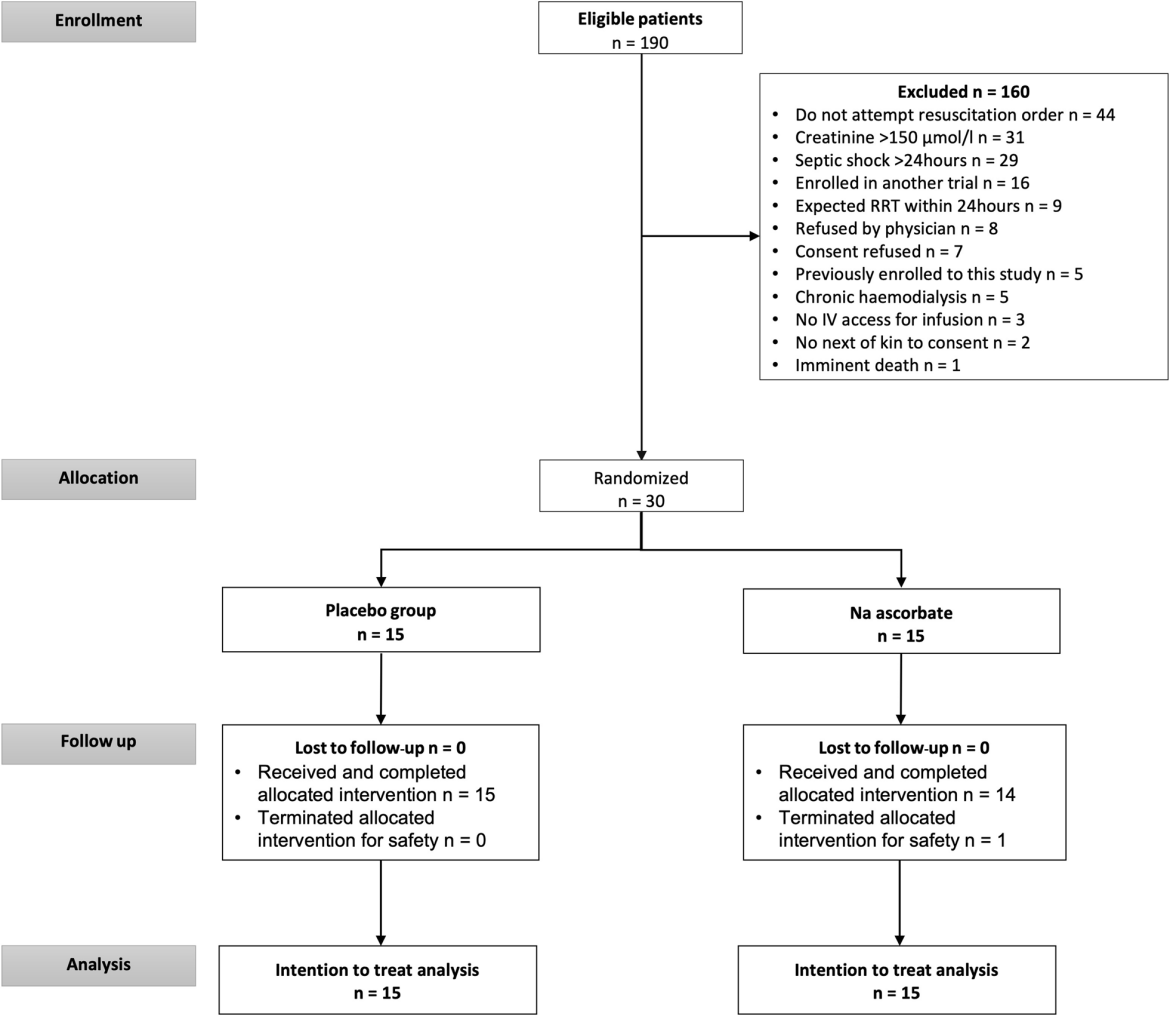
Trial screening and randomization flow chart

From October 28, 2020, to November 31, 2022, we screened 190 patients and randomized 30 (Fig. 1). Table 1 shows their baseline characteristics according to treatment group and indicates comparable illness severity scores. Except for one patient in the placebo group, who was only on metaraminol at randomization, all patients received norepinephrine at enrolment. The median (IQR) norepinephrine equivalent dose was 0.09 [0.06–0.18] µg/kg/min in the NaAscorbate group versus 0.06 [0.03–0.08] µg/kg/min in the placebo group. Six patients in the control group and 10 patients in the NaAscorbate group received hydrocortisone before enrolment. However, during trial drug infusion, an additional two patients in the control group received hydrocortisone (total of eight patients) whereas no additional patients in the NaAscorbate group did (total of 10 patients).

**Table 1:** Baseline characteristics of study patients

	Placebo (*n* = 15)	Na ascorbate (*n* = 15)
Male—no. (%)	9 (60)	12 (80)
Age—years	70 (56–79)	64 (48–71)
Body mass index—kg/m^2^	29.4 (24.2–32.6)	30.5 (24.9–36.9)
ICU admission source—no. (%)		
Emergency department	6 (40)	7 (47)
Operating or recovery room	6 (40)	4 (27)
Ward	2 (13)	1 (7)
Transfer from another hospital	1 (7)	3 (20)
Non-operative admission—no. (%)	8 (53)	11 (73)
Diabetes mellitus—no. (%)	4 (27)	4 (27)
Chronic kidney disease—no. (%)	1 (7)	1 (7)
APACHE III†	57 (54–62)	59 (43–82)
SOFA score‡	7 (5–8)	7 (6–8)
Sepsis source—no. (%)		
Gastro-intestinal/biliary	7 (47)	4 (27)
Primary bacteraemia	1 (7)	0 (0)
Respiratory	3 (20)	5 (33)
Soft tissue	2 (13)	0 (0)
Urinary	1 (7)	3 (20)
Other	1 (7)	3 (20)
Nosocomial infection—no. (%)	1 (7)	3 (20)
Serum lactate—mmol/L	1.6 (1.2–2.1)	2.1 (1.8–2.4)
White blood cells count—× 10^9^/L	9.6 (5.8–11.7)	11.0 (9.1–13.2)
Platelets count—× 10^9^/L	170 (147–233)	130 (83–184)
Serum creatinine—µmol/L	98 (87–128)	90 (64–128)
Respiratory support—no. (%)		
Mechanical ventilation	7 (47)	7 (47)
Non-invasive ventilation	1 (7)	0 (0)
High Flow Nasal Cannula	0 (0)	3 (20)
Nasal or room air	7 (47)	5 (33)
Hemodynamic support—no. (%)		
Norepinephrine alone	11 (73)	10 (67)
Norepinephrine + Vasopressin	2 (13)	5 (33)
Norepinephrine + Epinephrine	1 (7)	0 (0)
Metaraminol alone	1 (7)	0 (0)
Norepinephrine equivalent—µg/kg/min§	0.06 (0.03–0.08)	0.09 (0.06–0.18)
Hydrocortisone before enrolment—no. (%)	6 (40)	10 (78)
Hydrocortisone use from pre-enrolment to end of study drug infusion—no. (%)	8 (53)	10 (78)
Urine output at randomization—ml/hr	60 (40–103)	100 (58–145)
Furosemide use in the 6 h before commencement of study drug—no. (%)	2 (13)	1 (7)
Furosemide use from start to 24 h after commencement of study drug—no. (%)	7 (47)	7 (47)

Numerical values are presented as median [IQR]. ICU denotes intensive care unit

^†^Acute physiology and chronic health evaluation (APACHE) III score

^‡^Sequential organ failure assessment (SOFA) score

^§^Vasopressin, epinephrine, metaraminol and angiotensin has been converted using the appropriate formula (Additional file 1: Supplementary Appendix)

### Primary outcome

**Fig. 2:**
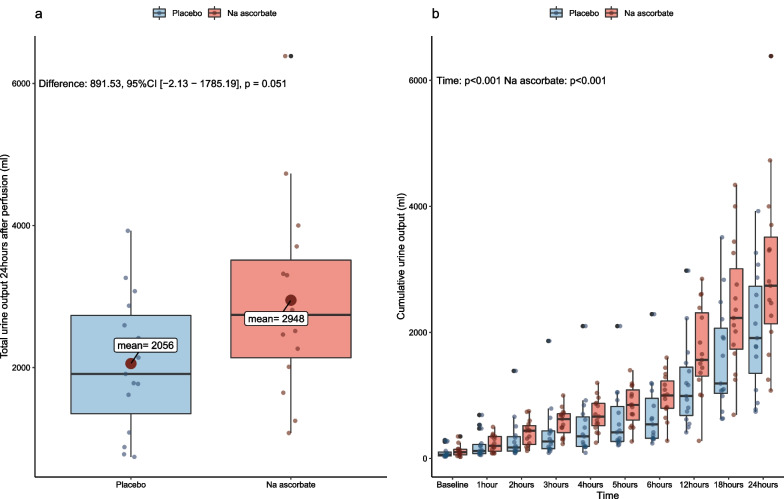
Panel **a**: Total urine output 24 h after start of trial drug infusion. Panel **b** Progressive cumulative urinary output over time

The mean (95% confidence interval [CI]) total UO at 24 h was 2948 (2181–3715) ml with NaAscorbate compared to 2056 (1520–2593) ml with placebo, an 891 ml difference (95% CI − 2 to 1785 ml, *P* = 0.051) (Fig. 2a and Table 2). Moreover, on linear mixed modelling, the increase in UO over time was significantly greater with NaAscorbate (*P* < 0.001) (Fig. 2b).

**Table 2:** Study outcomes

Outcome	Placebo (*N* = 15)	Na ascorbate (*N* = 15)	Difference (95% CI)
	Mean (95% CI)	Mean (95% CI)	
*Primary outcome*			
Total urine output at 24 h (ml)	2056 (1520–2593)	2948 (2181–3715)	891.5 (− 2.1 to 1785.2) ||

	Median (IQR)	Median (IQR)	Difference (BCa 95% CI)
*Secondary outcomes*			
Alive and vasopressor-free hours 7 days	151 (124–160)	148 (136–162)	3 (− 26 to 19)
Alive and ICU-free days at day 28	23 (21–26)	21 (17–25)	2 (− 3 to 6)
Hospital length of stay (day)	11 (8–24)	15 (8–29)	− 4 (− 23 to 8)
Alive and ventilator-free hours at 28 days	637 (563–659)	605 (482–652)	32 (− 103 to 250)
Peak serum creatinine at day 7 (µmol/L)	111 (89–134)	96 (69–195)	15 (− 79 to 59)
Time from randomization to start of study drug (hours)	0.58 (0.38–0.87)	0.92 (0.71–1.21)	− 0.34 (− 0.75 to 0)
Time from sepsis diagnosis to start of study drug (hours)	14 (10, 21)	11 (6–14)	3 (− 4 to 10)
Incidence of hypernatremia	0 (0)	1 (7)	–

	No. of patients (%)	No. of patients (%)	Difference (95% CI)
Death before ICU discharge by day 28	1 (7)	3 (20)	− 13 (− 30 to 4)
Death before discharge home by day 28	1 (7)	3 (20)	− 13 (− 30 to 4)
Death from any cause at any location by day 28	1 (7)	3 (20)	− 13 (− 30 to 4)
Renal replacement therapy	0 (0)	2 (13)	–

|| *P* = 0.051

BCa = bias-corrected 95% confidence interval

### Feasibility, sodium, creatinine, and oxalate

**Fig. 3:**
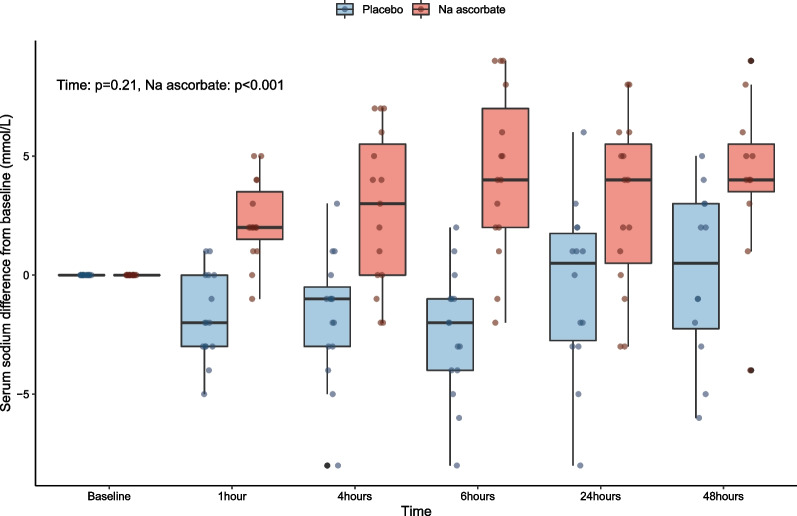
Serum sodium changes from baseline over time

Study treatment was not completely delivered in one patient because of a peak sodium level of 161 mmol/L and falsely elevated ketone levels by point of care device after 50 g of the trial drug infusion. Consistent with this observation, the mixed linear regression model showed a significantly higher serum sodium change from baseline over time with NaAscorbate (*P* < 0.001) (Fig. 3). The peak of serum creatinine to day seven was comparable between groups (Table 2). No urinary oxalate crystals were seen in either group (Table 2).

### Ascorbate levels and secondary outcomes

**Fig. 4:**
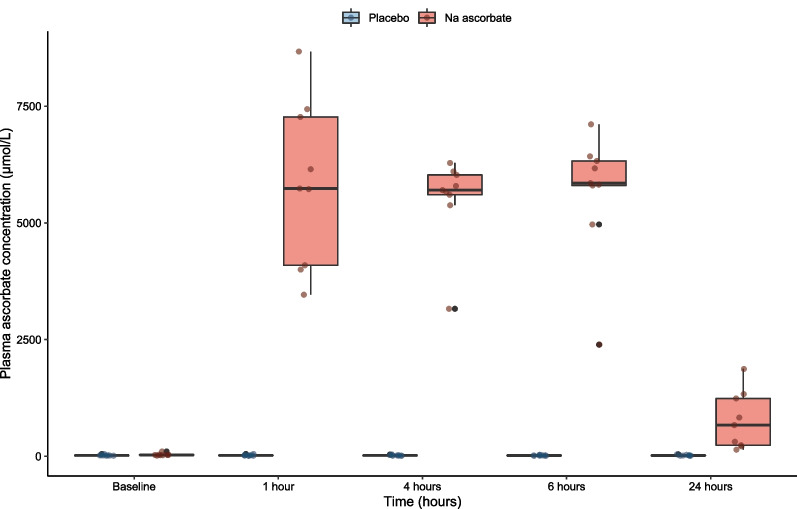
Plasma ascorbate concentration during the first 24 h

We collected blood samples from 18 patients (from the 1st patient to 18th patient). As shown in Fig. 4, at baseline, plasma ascorbate concentrations were low overall and eight of the study patients fulfilled the criteria for vitamin C hypovitaminosis with median baseline values of 19 µmol/L in the placebo group and 30 µmol/L in the NaAscorbate group (Additional file 1: Table S1). One hour after the loading dose of the study drug, however, the plasma ascorbate concentration increased to a median [IQR] of 5736 [4093–7270] µmol/L. It was then stable at such level during the 6 h of maintenance infusion and remained more than 20 times higher than baseline at 24 h (Additional file 1: Table S1). In contrast, plasma ascorbate concentrations did not change from baseline in the placebo group.

**Fig. 5:**
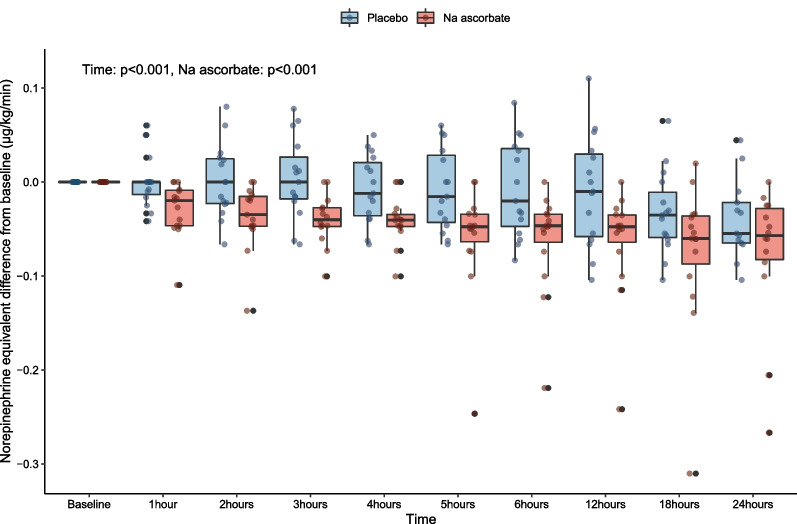
Norepinephrine equivalent changes from baseline to 24 h

**Fig. 6:**
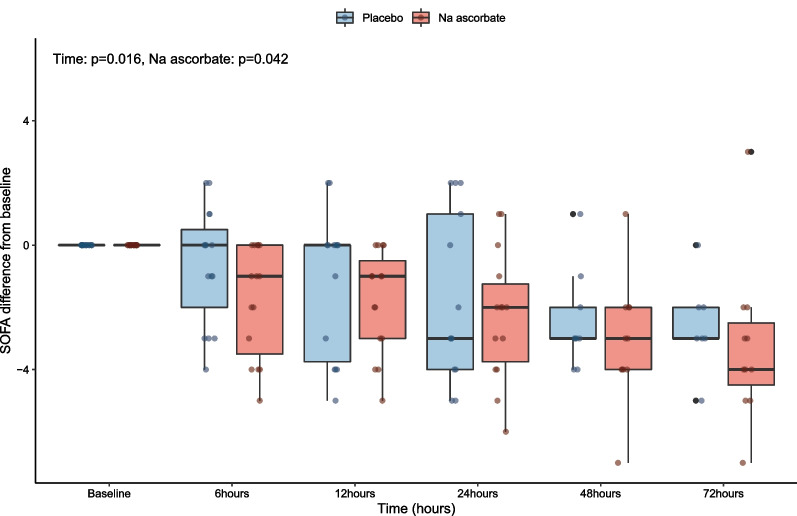
SOFA score changes from baseline to 72 h

Compared with placebo, the decrease in vasopressor dose over time was significantly different with a − 0.04 µg/kg/min greater decrease over time (*P* < 0.001) with NaAscorbate (Fig. 5). The change in SOFA score over time within the first 72 h was also significantly greater (*P* = 0.042) (Fig. 6).

### Inflammatory markers analysis

At baseline, the plasma concentration of VEGFα, IL-10, IL-6 and IL-8 were similar between groups (Additional file 1: Fig. S3). The baseline plasma CRP concentration was numerically higher in the NaAscorbate group at a median of 607 mg/L compared with 308 mg/L in the control group. The temporal changes, however, were similar between the two groups with a significant decrease for IL-6 and IL-8 in both groups (*P* < 0.001 and *P* = 0.0012, respectively) (Additional file 1: Figs. S3 and S4).

### Biochemical outcomes analysis

NaAscorbate affected acid base balance with a modest but significantly greater (*P* = 0.021) increase in serum bicarbonate than placebo over time on linear mixed regression modelling (Additional file 1: Fig. S4). It also led to a concordantly significantly greater (*P* = 0.0029) increase in base excess than placebo (Additional file 1: Fig. S4).

### Adverse events

One patient with chronic kidney disease developed hypernatremia (Na of 161 mmol/L) and a falsely elevated ketone level by point of care device after 50 g of NaAscorbate infusion. Such hypernatremia resolved over 24 h after cessation of the study drug. All glucose levels were measured by blood gas machine and there was no false elevated glucose report. Another patient developed evidence of moderate hemolysis 12 h after NaAscorbate administration had been completed. This occurred in the setting of a large intraperitoneal hematoma being reabsorbed and was accompanied by an increase in methemoglobin. Independent hematological assessment was that the patient had probable NaAscorbate-dependent oxidative stress induced hemolysis. The patient received a blood transfusion and the hemolysis resolved spontaneously over 48 h. The patient was discharged alive from ICU with a stable hemoglobin level and no further evidence of hemolysis after radiological drainage of the hematoma. The patient did not have G6PD deficiency. Both episodes were immediately reported to the Austin Health Human Research Ethics Committee.

## Discussion

### Main findings

In this double-blind, randomized, controlled study in septic shock patients, we compared the physiological effects of a single 60-g dose of NaAscorbate infused over 6 h with placebo. We found that the difference in total 24-h urine output was not statistically different. However, NaAscorbate induced a greater cumulative increase in urine output over time. Moreover, it led to a greater decrease in vasopressor dose and SOFA score over time. Finally, we showed a dramatic effect on plasma ascorbate levels but no significant effect on biomarkers of inflammation.

### Relationship with previous studies

Our findings appear to differ from those of recent randomized controlled trials, which assessed the effect of very high dose vitamin C in patients with sepsis [10, 12]. In the Lessening Organ Dysfunction With VITamin C (LOVIT) trial and the Vitamin C Infusion for Treatment in Sepsis Induced Acute Lung Injury (CITRIS-ALI) trial, 50 mg/kg of ascorbic acid (4 g in an 80 kg person) or placebo was given to patients every 6 h. In the LOVIT trial, the risk of death or persistent organ dysfunction, including vasopressor use, at 28 days was higher in the vitamin C group than in the placebo group and urine output was similar between groups [12]. In the CITRIS-ALI trial, the same infusion regimen was given to patients with sepsis and acute respiratory distress syndrome (ARDS) and did not significantly improve organ dysfunction scores or alter markers of inflammation and vascular injury [10]. In addition, a lack of effect was also seen in another randomized trial in septic shock patients where vitamin C was combined with hydrocortisone and thiamine [11]. Similarly, another study, that administered a one gram bolus of ascorbic acid followed by 250 mg/hr of continuous infusion for 96 h to patients with sepsis, demonstrated a higher renal replacement therapy rate in the ascorbic acid group [23]. Finally, current guidelines recommend not to use intravenous vitamin C in patients with sepsis because of possible harm [13].

A possible explanation for our findings might relate to the amount of the study drug administered. In our study, for example, the median serum vitamin C (measured as ascorbate) concentration was approximated 6000 µmol/L. This is more than 30 times higher than in the CITRIS-ALI trial [10]. Achieving very high blood levels might affect the capacity of ascorbate to affect biological pathways as suggested by a recent systematic review [24–26], and as shown in experimental studies, where complete resolution of sepsis was achieved in a few hours with ascorbate concentrations of approximately 10,000 µmol/L [16].

Another possible explanation may relate to the preparation used. In our trial, we used NaAscorbate, which has a physiological pH. In contrast, in the LOVIT trial, investigators administered ascorbic acid [12]. As recently described in detail [27], the preparation used in the LOVIT trial has a pH range between 5.4 and 5.6 and can induce significant metabolic acidosis and acidemia in experimental animals. In contrast, NaAscorbate at the same dose maintains a physiological pH. A similar effect may have occurred in septic humans and may explain the differential findings of the LOVIT trial (and possibly other trials using ascorbic acid as well) and those of our pilot study.

Finally, single mega-dose vitamin C therapy has already been given to patients in conditions other than septic shock with doses > 200 g/day [28]. Such treatment has been mostly in the setting of adjuvant therapy for cancer and showed no differential increases in adverse events compared with placebo. More work, however, is needed to establish whether such a safety profile applies to septic shock patients as well.

### Implications of study findings

Our findings imply that 60 g of Na Ascorbate given over 6 h significantly increases UO over time compared with placebo. Moreover, these observations suggest that such treatment can also lead to a greater decrease in vasopressor dose and SOFA score over time than placebo. Finally, they suggest that such mega-dose treatment can deliver ascorbate blood levels which are up to 30 times those seen in a previous trial of high dose therapy [12], without widespread signs of toxicity, or the induction of acidosis. However, the lack of changes in inflammatory mediators suggests little or no effect of NaAscorbate on the cytokine response to sepsis. Although our case of hemolysis may be idiosyncratic as suggested by a scoping review [28] or even spurious given the clinical scenario, mega-dose NaAscorbate therapy may not be free of risks. Thus, monitoring of sodium and hemoglobin levels and cessation of drug infusion if significant deviations occur is vital.

### Strengths and limitations

Our study has several strengths. It is the first, double-blind, randomized controlled trial to assess the physiological effects of a single mega-dose of NaAscorbate in patients with septic shock. All patients were enrolled within 24 h of onset suggesting that they were in the acute phase of septic shock. We measured the plasma concentration of ascorbate and demonstrated that, in relation to such levels, our treatment profoundly differed from previous trials using vitamin C as a therapy. Finally, we used a preparation which, unlike ascorbic acid-based preparations, has physiological pH levels and, therefore, did not induce acidemia and indeed delivered a mild degree of alkalosis.

We acknowledge some limitations. First, we amended the protocol after the first 12 patients. However, this was a pilot feasibility and physiological effect trial, the equivalent of first in man for this intervention in septic shock, and safety was paramount. Second, the recruitment rate was low and profoundly affected by the COVID pandemic. Third, the physiological effects seen are not necessarily due to ascorbate and may simply reflect the impact of a sodium load. In addition, we enrolled patients without CKD and most of the patients received norepinephrine at enrolment, which implies a cardiovascular SOFA score of 3 or more. Thus, the change in vasopressor therapy was responsible for the change in SOFA score. Finally, this trial is a small single-center study and does not have enough power to provide significant information about clinical outcomes.

## Conclusion

In a pilot, double-blind, randomized, and controlled study of septic shock patients, a single mega-dose of NaAscorbate increased cumulative urine output, decreased vasopressor requirements, and lowered SOFA scores over time. Moreover, it markedly increased plasma ascorbate to 30-fold greater levels than previously reported in critically ill patients. Finally, it achieved these physiological changes without inducing acidosis or urinary oxalate crystal formation. However, it also modestly increased sodium levels and did not lower the levels of inflammatory biomarkers. Also, NaAscorbate may have contributed to hypernatremia and hemolysis in one patient. Further cautious investigations of this novel intervention appear justified.

### Supplementary Information

**Additional file 1**. Supplementary Appendix.

## Data Availability

The datasets used and/or analyzed during the current study are available from the corresponding author on reasonable request.
