# Supplementary material for: Mega-dose sodium ascorbate: a pilot, single-dose, physiological effect, double-blind, randomized, controlled trial
Source: Crit Care. 2023 Oct 12;27:371. doi: 10.1186/s13054-023-04644-x (PMC10571252; doi:10.1186/s13054-023-04644-x)

**Supplementary Appendix**

Initial exclusion criteria (From the 1^st^ to 12^th^ patient)

***Exclusion criteria***

Patients will be EXCLUDED from the study if ONE of the following criteria presents:

1. Age < 18 years
2. Pregnancy
3. Do not resuscitate/Do not intubate (DNR/DNI) orders.
4. Death is deemed to be imminent or inevitable during this admission, and either the attending physician, patient or substitute decision-maker is not committed to active treatment.
5. Patients with known HIV infection
6. Patients with known glucose-6 phosphate dehydrogenase (G-6PD) deficiency
7. Patients transferred from another ICU or hospital with a diagnosis of a septic shock for > 24 hours.
8. Patients with a diagnosis of a septic shock for > 24 hours
9. Patients with known or suspected
   1. History of oxalate nephropathy or hyperoxaluria
   2. Short bowel syndrome or severe fat-malabsorption
   3. Malaria
   4. Scurvy
10. Patient previously enrolled in this study.
11. Patients with chronic haemodialysis or peritoneal dialysis.
12. Patients require renal replacement therapy within next 24 hours.
13. Patient’s baseline blood sodium level was >160 mmol/L.

***Revised exclusion criteria (from 13^th^ patient)***

1. Age < 18 years
2. Pregnancy
3. Death is deemed to be imminent or inevitable during this admission, and either the attending physician, patient or substitute decision-maker is not committed to active treatment.
4. Patients with known HIV infection
5. Patients with known glucose-6 phosphate dehydrogenase (G-6PD) deficiency
6. Patients transferred from another ICU or hospital with a diagnosis of a septic shock for < 24 hours.
7. Patients with a diagnosis of a septic shock for < 24 hours
8. Patients with known or suspected
   1. History of oxalate nephropathy or hyperoxaluria
   2. Short bowel syndrome or severe fat-malabsorption
   3. Malaria
   4. Scurvy
9. Patient previously enrolled in this study.
10. Patients with chronic haemodialysis or peritoneal dialysis.
11. Patients require renal replacement therapy within next 24 hours.
12. Patient’s baseline blood sodium level is < 155 mmol/L.
13. Patient’s creatinine at enrolment is ≤ 150 µmol/L.

Vasopressor formula for conversion to norepinephrine equivalent dose

| Drug | Dose | Norepinephrine equivalent |
| --- | --- | --- |
| Epinephrine | 0.1 µg/kg/min | 0.1 µg/kg/min |
| Norepinephrine | 0.1 µg/kg/min | 0.1 µg/kg/min |
| Vasopressin | 0.04 U/min | 0.1 µg/kg/min |
| Metaraminol | 1.0 µg/kg/min | 0.1 µg/kg/min |

**Supplemental Table S1: Median (IQR) Na ascorbate concentration (µmol/L) at each study time points.**

|  | Placebo | Na ascorbate |
| --- | --- | --- |
| Baseline | 19 [16-23] | 30 [25-35] |
| 1H | 20 [15-24] | 5736 [4093-7270] |
| 4H | 20 [14-23] | 5703 [5603-6028] |
| 6H | 18 [13-24] | 5853 [5802-6327] |
| 24H | 18 [14-25] | 669 [237-1240] |

**Supplemental Figure S1: Normality of urine output at 24h by (a): histogram of the distribution among the groups and (b): Q-Q plot with Shapiro-Wilk’s Test**


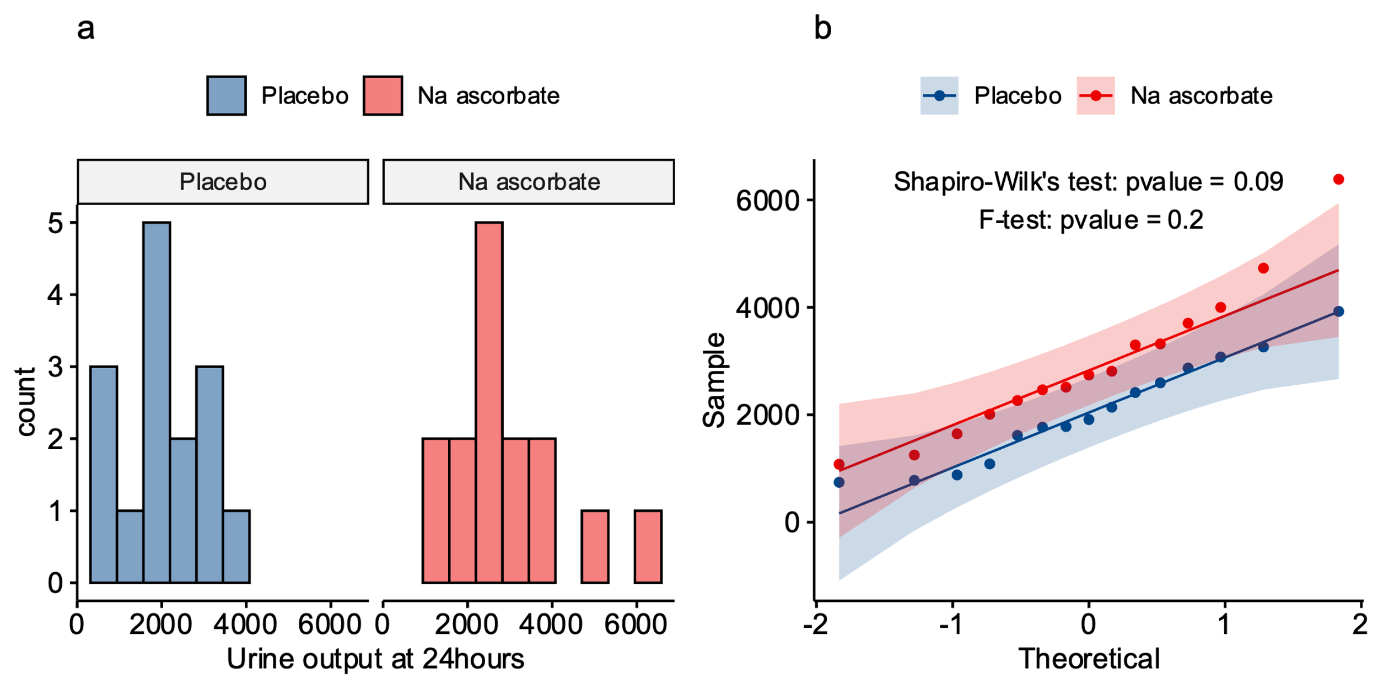


**Supplemental Figure S2: Linear mixed model histogram and QQ-plot of the residual distribution for (a): cumulative urine output; (b): Sodium evolution; (c): Norepinephrine equivalent evolution; (d): SOFA score evolution**

**Supplemental Figure S3: Inflammatory biomarkers serum concentration expressed as difference from baseline (left) or absolute measurements (right) for (a) VEGF-α; (b) IL-10; (c) IL-6; (d) IL10 and (e) C-Reactive Protein. The linear mixed models have been performed only for the changes from baseline.**


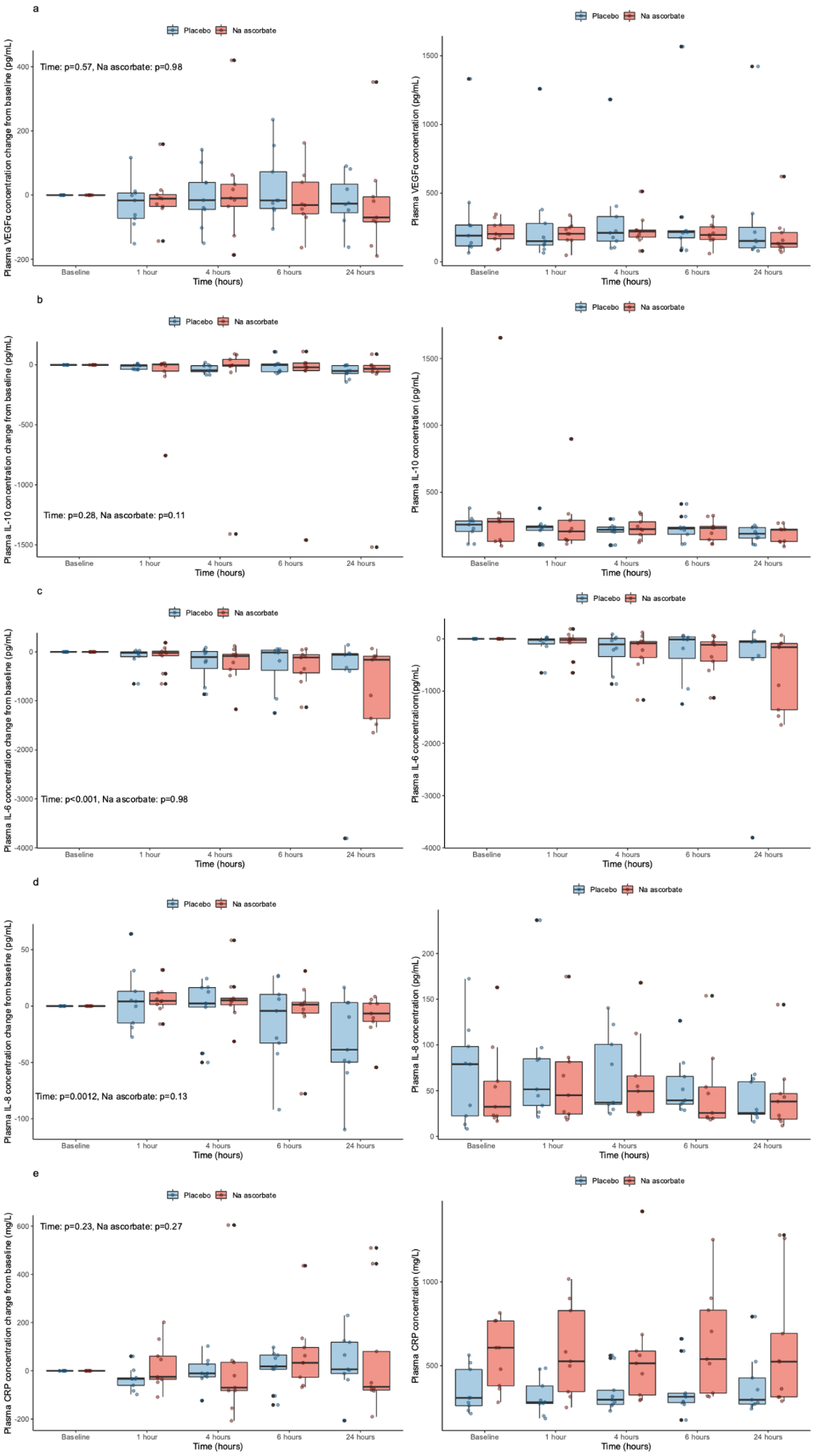


**Supplemental Figure S4: Linear mixed model histogram and QQ-plot residuals distribution for (a): Log plasma Na ascorbate change, (b): Plasma VEGF-α change, (c) Plasma IL-10 change, (d) Plasma IL-5 change, (e) Plasma IL-8 change, (f) Plasma CRP change.**

**Supplemental Figure S4: Arterial blood gas analysis expressed as difference from baseline (left) or absolute measurements (right) for pH, Bicarbonate and Base excess. The linear mixed models were performed only for the changes from baseline.**


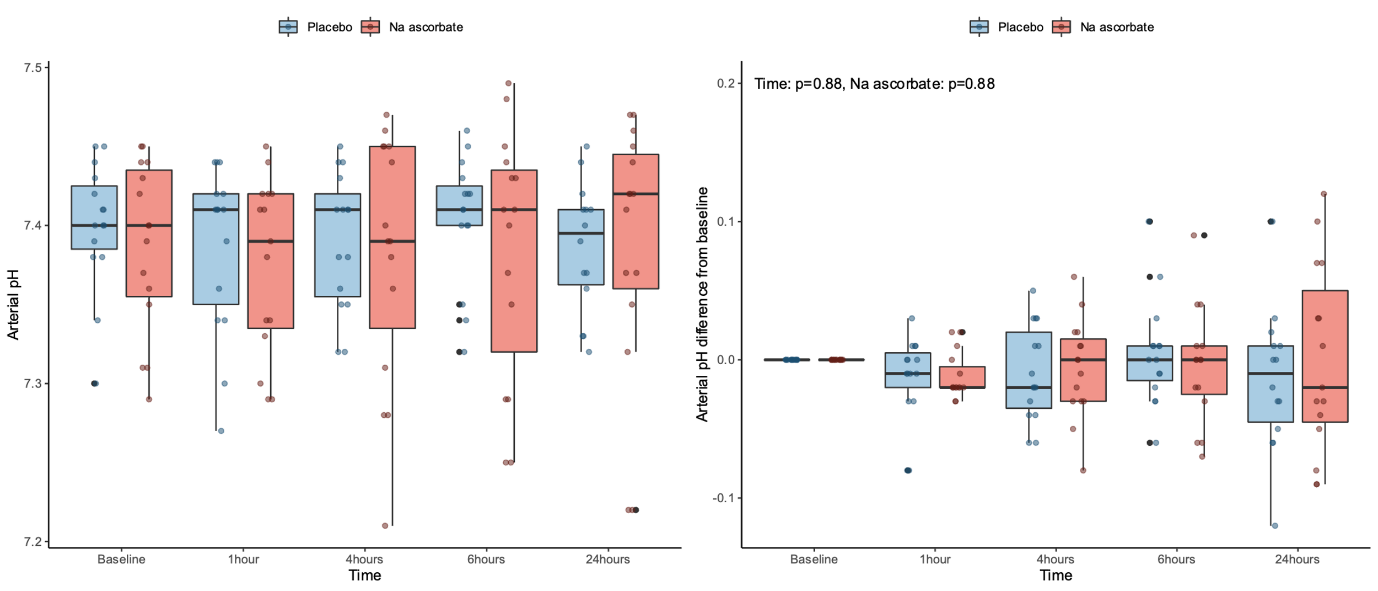


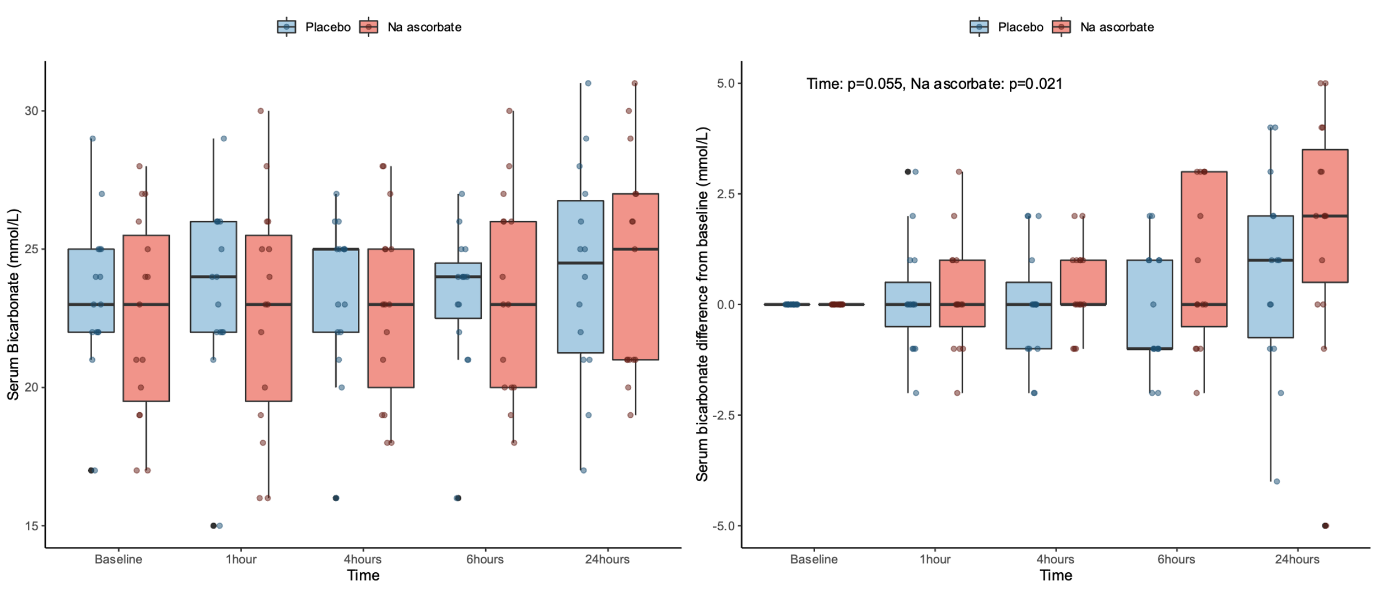


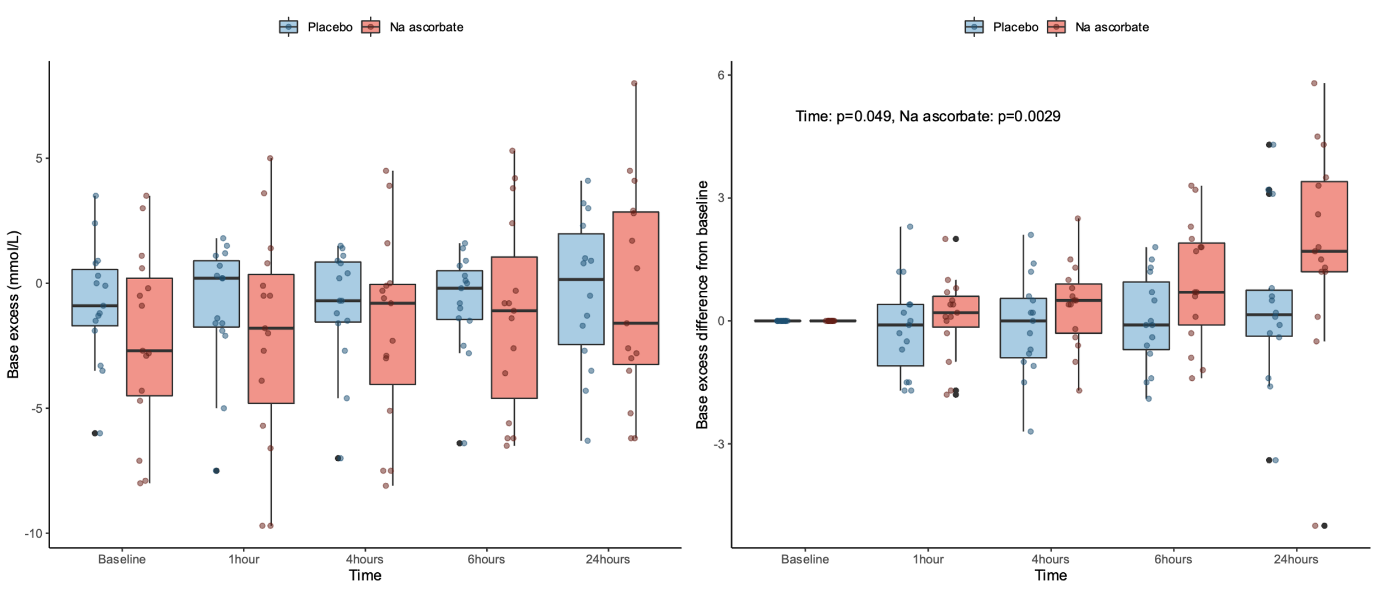

Supplement: Supplementary file 1 — Additional file 1. Supplementary Appendix. [file 13054_2023_4644_MOESM1_ESM.docx]
